# Hypochondriasis

**Published:** 1830-04-01

**Authors:** 


### XXXIII.

### Hypochondriasis.

We are apt to believe a merry companion the happiest fellow in the world, and envy him, perhaps, his light heart and airy spirits; but such men have hours of melancholy, when the spirits sink, and a gloom comes over them, deeper and darker than is ever known to their less excitable companions. A man may be cheerful on paper, though he has a heavy heart, and brilliant in company, though insufferably wretched when left to commune with his own soul.

The extremes of low and high spirits, which occur in the same person at different times, are happily illustrated by the following case, related by Dr. Rush:—" A physician, in one of the cities of Italy, was once consulted by a gentleman who was much distressed by a paroxysm of this intermitting state of hypochondriacism. He advised the melancholy man to seek relief in convivial company, and recommended him in particular to find out a celebrated wit by the name of Cardini, who kept all the tables of the city, to which he was invited, in a roar of laughter, and to spend as much time with him as possible. 'Alas! Sir,' said the patient, with a heavy sigh, 'I am that Cardini.' "

---

